# Primary Human Alveolar Bone Cells Isolated from Tissue Samples Acquired at Periodontal Surgeries Exhibit Sustained Proliferation and Retain Osteogenic Phenotype during *In Vitro* Expansion

**DOI:** 10.1371/journal.pone.0092969

**Published:** 2014-03-25

**Authors:** Darja Marolt, Matjaz Rode, Nevenka Kregar-Velikonja, Matjaz Jeras, Miomir Knezevic

**Affiliations:** 1 Blood Transfusion Center of Slovenia, Ljubljana, Slovenia; 2 Educell d.o.o., Trzin, Slovenia; 3 Community Health Center, Ljubljana, Slovenia; 4 Faculty of Health Sciences, Novo mesto, Slovenia; 5 Faculty of Pharmacy, University of Ljubljana, Ljubljana, Slovenia; 6 Celica d.o.o. Biomedical Centre, Ljubljana, Slovenia; Johns Hopkins University, United States of America

## Abstract

**Objectives:**

Bone tissue regeneration requires a source of viable, proliferative cells with osteogenic differentiation capacity. Periodontal surgeries represent an opportunity to procure small amounts of autologous tissues for primary cell isolation. Our objective was to assess the potential of human alveolar bone as a source of autologous osteogenic cells for tissue engineering and biomaterials and drug testing studies.

**Materials and Methods:**

Alveolar bone tissue was obtained from 37 patients undergoing routine periodontal surgery. Tissue harvesting and cell isolation procedures were optimized to isolate viable cells. Primary cells were subcultured and characterized with respect to their growth characteristics, gene expression of osteogenic markers, alkaline phosphatase activity and matrix mineralization, under osteogenic stimulation.

**Results:**

Alveolar bone cells were successfully isolated from 28 of the 30 samples harvested with bone forceps, and from 2 of the 5 samples obtained by bone drilling. The yield of cells in primary cultures was variable between the individual samples, but was not related to the site of tissue harvesting and the patient age. In 80% of samples (n = 5), the primary cells proliferated steadily for eight subsequent passages, reaching cumulative numbers over 10^10^ cells. Analyses confirmed stable gene expression of alkaline phosphatase, osteopontin and osteocalcin in early and late cell passages. In osteogenic medium, the cells from late passages increased alkaline phosphatase activity and accumulated mineralized matrix, indicating a mature osteoblastic phenotype.

**Conclusions:**

Primary alveolar bone cells exhibited robust proliferation and retained osteogenic phenotype during *in vitro* expansion, suggesting that they can be used as an autologous cell source for bone regenerative therapies and various *in vitro* studies.

## Introduction

Bone regeneration requires a source of viable, proliferative cells with osteogenic differentiation capacity. The cells can either be stimulated to migrate from the neighboring tissue, or delivered to the defect site by transplantation of autologous or heterologous bone grafts or tissue-engineered (TE) bone substitutes [1], [2], [3]. A number of bone tissue engineering approaches are being investigated, where osteogenic cells, responsible for the synthesis, organization and remodeling of the new bone tissue, are combined with scaffolding materials – structural and logistic templates for cell attachment and tissue development, and growth factors - bioactive cues that mediate the cell activity [4], [5], [6]. In cases where the quantity of autologous bone tissue for transplantation is limited, implantation of viable TE-bone substitutes represents an alternative to enhance the process of bone repair [7]. In addition, development and testing of new drugs and biomaterials could benefit from using physiologically relevant human cell models, to evaluate the effects on specialized cell survival and activity [8]. For instance, recent reports of osteonecrosis of the jaw, which were associated with the use of bisphosphonates, suggest the importance of drug testing directly in tissue-specific human cell models [9], [10], [11].

Human osteogenic cells can be isolated from various adult tissues, including bone, bone marrow, periosteum and adipose tissue [12], [13], [14], [15]. Previous studies have indicated differences in cell yields, proliferation and osteogenic potentials between these sources [16], [17]. Also, the influences of tissue harvesting and cell isolation procedures on the cell yields and phenotypes were observed [18], [19], [20], [21]. For the preparation of TE-bone substitutes, relatively large cell numbers are needed (millions to billions), and careful selection of harvesting and *in vitro* culture conditions can significantly increase the cell yields and improve the retention of osteogenic potential [21], [22], [23].

Ideally, autologous cells should be used for bone tissue engineering, to avoid the risks of immune rejection and infectious disease transmission. Consequently, availability of the source tissue for cell isolation and the invasiveness of harvesting procedures, which can result in donor site morbidity, represent important considerations. Periodontal surgical procedures, such as the placement of dental implants, represent an opportunity to procure small amounts of remaining autologous bone tissue for cell isolation, without causing additional injury to the patients. Previous studies indicate that alveolar bone can be used to isolate cells expressing characteristic mesenchymal surface markers, which have the potential to undergo osteogenic differentiation in appropriate culture conditions [12], [24], [25], [26], [27]. Furthermore, TE-constructs prepared from alveolar bone cells were shown to enhance *de novo* bone formation in critical-size skull defects in immunodeficient mice [26], [28], and were more recently used to treat jaw bone defects in several clinical case studies [29], [30], [31]. Importantly, prior work also suggests that osteogenic cells originating from the jaw bone exhibit distinct differentiation properties *in vitro* and *in vivo* [32], as well as distinct drug responses compared to osteogenic cells originating from the long/iliac bones [10], [11]. These studies clearly indicate the existence of physiological differences between bone cell populations at different anatomic locations. Therefore, primary human alveolar bone cells might be uniquely suited for periodontal and maxillofacial tissue engineering-based bone repair, and could represent a physiologically relevant model for *in vitro* studies related to periodontal treatment and regeneration. However, compared to primary bone cells from other anatomical locations, the effects of isolation and *in vitro* culture conditions on the properties of primary alveolar bone cells, which can significantly affect their clinical potential and the outcomes of bone regeneration treatments, are largely unknown.

For the purposes of *in vitro* studies, as well as for future clinical translation, it is thus necessary to evaluate the harvesting and expansion reproducibility of primary alveolar bone cells, obtained from a number of different patients, and to characterize the maintenance of osteogenic potential/phenotype during extended *in vitro* cultivation. Therefore, the aim of our work was to assess the parameters of human alveolar bone harvesting at the time of periodontal surgical procedures, and to evaluate the properties of isolated cells during *in vitro* expansion. Using bone samples from 37 patients, we optimized the tissue harvesting and cell isolation procedures, and quantitatively compared the cell yields and the osteogenic phenotype expression during *in vitro* culture. We found no effects of patient age, tissue harvesting site and cell isolation procedures on the cell yields and osteogenic marker expression. We found that alveolar bone cells exhibit robust proliferation in the absence of growth factor supplementation, and retain their osteogenic phenotype during *in vitro* expansion. Our data suggests that primary alveolar bone cells can be used as a physiologically relevant *in vitro* study model, as well as a source of autologous cells for the preparation of TE-bone substitutes.

## Materials and Methods

### Ethics statement

The patients gave verbal consent for the use of discarded tissues for research purposes. The patient consents were included with the samples. Tissue samples were deidentified and analyzed anonymously. The study was approved by the National Medical Ethics Committee (approval number 74/05/03).

### Material

Dulbecco's Modified Eagle's Medium/Nutrient Mixture F-12 Ham 1:1 (DMEM/F12) and Fetal Bovine Serum (FBS) were from Lonza (East Rutherford, NJ). Fungizone, gentamicin and trypsin/ethylenediaminetetraacetic acid (EDTA) were from Life Technologies (Carlsbad, CA). Ascorbic acid 2-phosphate, dexamethasone, β-glycerophosphate, collagenase, TRI Reagent and silver nitrate were from Sigma-Aldrich (St. Louis, MO). Random primers were purchased from Promega (Madison, WI). All other chemicals were of analytical or pharmaceutical grade and were obtained from Sigma-Aldrich.

### Human tissue harvesting

Samples of remaining alveolar bone tissues were obtained from the maxillae or mandibles of 37 patients undergoing periodontal surgical procedures. In all experiments, tissue samples from different patients were kept separately and the isolated cells were cultured separately. Bone tissue was harvested by either using bone forceps or bone drilling, and transferred into fresh DMEM/F12 supplemented with 50 μg/ml gentamicin and 0.08% vol/vol Fungizone. Bone sample characteristics are summarized in **Table 1**. Bone tissue was cut into small pieces (0.5–1 mm diameter), thoroughly washed with DMEM/F12 supplemented with 50 μg/ml gentamicin and 0.08% vol/vol Fungizone and checked under the microscope for the removal of adjacent bone marrow tissue. Due to small amounts of harvested tissue samples, their total volumes were assessed by comparison with a set of standards and carefully noted for each sample. Each tissue sample was then processed for cell isolation using one of the two or both cell isolation procedures described below.

**Table 1:** Characteristics of alveolar bone samples, tissue harvesting and cell isolation procedures used in the study.

Alveolar bone samples	37 total			
Site of tissue harvest	26 maxilla, 11 mandible			
Patient gender	22 female, 15 male			
Patient age	23–74 years, average 53 years			
Quantity of tissue	2–400 mm^3^ tissue, average 64 mm^3^ tissue			
**Tissue acquisition**	7 samples bone drilling		30 samples bone forceps	
Number plated/location	2 maxilla	5 mandible	24 maxilla	6 mandible
Number growing/location	2 maxilla	0 mandible*	23 maxilla	5 mandible
Total growing	29% (2/7)		93% (28/30)	
**Cell yield analyses**	20 samples explants		18 samples collagenase	
Number plated/location	16 maxilla	4 mandible	14 maxilla	4 mandible

*In failed mandibular samples, both explant cultures and collagenase digestion were tested for the preparation of primary cultures.

### Alveolar bone cell isolation and culture

Based on previous reports [12], [21], two procedures for cell isolation were tested (Table 1). For the preparation of explant cultures (n = 20), ∼20 mm^3^ aliquots of the bone tissue were transferred into each well of the 6-well cell culture plates. In case of enzymatic tissue digestion (n = 18), ∼50 mm^3^ of washed tissue pieces were transferred per each 15 ml centrifuge tube containing 5 ml collagenase solution (DMEM/F12 with 1 mg/ml collagenase, 50 μg/ml gentamicin and 0.08% vol/vol Fungizone) at 37°C. The tubes were kept rotating for 30 min at 37°C, thoroughly vortexed and then left to settle. Supernatants were carefully removed, transferred to a new 15 ml centrifuge tube and the collagenase solution was neutralized with the addition of an equal volume of the culture medium (DMEM/F12 supplemented with 20% FBS, 50 μg/ml gentamicin and 0.08% Fungizone). We chose the same cell culture medium as in our previous studies [10], [25]. Fresh collagenase solution was added to the remaining osseous tissue and the digestion process was repeated 5-times. Supernatants from the first and the second digestion steps were discarded. Those from the following four digestion steps were poured on the remaining undigested tissue and centrifuged at 250 × g for 5 min. The resulting pellets were resuspended in the culture medium and seeded into 6-well dishes (∼20 mm^3^ of the initial bone tissue in 3 ml of the culture medium per well). The cultures were incubated at 37°C in a humidified atmosphere containing 5% CO_2_ in the air (standard conditions). After 3 days of undisturbed culturing, 1 ml of the culture medium was exchanged with fresh medium, followed by complete media changes twice per week. After reaching confluence, the cells were detached with the 0.02% vol/vol trypsin/EDTA solution, resuspended in culture medium and counted. Cell yields seeded in primary cultures were calculated by normalizing the total number of isolated cells in the sample with the sample volume.

Cells obtained by each of the two isolation procedures from 5 samples were cultured separately for eight sequential passages. At each passage, the cells were seeded into tissue culture flasks at a density of ∼5.000 cells/cm^2^, cultured to confluence (conf.), trypsinized, counted and re-passaged (re-pass.). The number of cell doublings and the increase in total cell numbers were calculated at each passage, taking into account the cells used for phenotype analyses. An estimate of the specific growth rate (μ) was calculated at each subculture step, as follows:
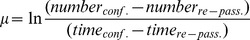



### Gene expression analyses

Gene expression of alkaline phosphatase (AP), osteopontin (OP) and osteocalcin (OC) were analyzed quantitatively in the second passage of cells isolated from 13 samples. Additionally, gene expression levels were monitored during subcultivation from the first to the fifth passage of 6 patient samples. Total RNA was extracted from the cells using 1 ml of TRI Reagent/10^6^ cells, according to the manufacturer's instructions (Sigma-Aldrich, St. Louis, MO). Approximately 1 μg of the total RNA was reverse-transcribed using random primers and the SuperScript II RNase H^−^ Reverse Transcriptase, following manufacturer's instructions (Life Technologies). Gene expression was quantified using ABI Prism 7900HT Real Time PCR system (Applied Biosystems/Life Technologies). The PCR reaction conditions were: 2 min at 50°C, 10 min at 95°C and 50 cycles at 95°C for 15 s and 1 min at 60°C. The following primers and probes (Applied Biosystems, Foster City, CA) were used [33]: AP forward primer 5′-GACCCTTGACCCCCACAAT-3′; AP reverse primer 5′-GCTCGTACTGCATGTCCCC-3′; AP probe 5′-TGGACTACCTATTGGGTCTCTTCGAGCCA-3′; OP forward primer 5′-CTCAGGCCAGTTGCAGCC-3′; OP reverse primer 5′- CAAAAGCAAATCACTGCAATTCTC -3′; OP probe 5′-AAACGCCGACCAAGGAAAACTCACTACC -3′; OC forward primer 5′-GAAGCCCAGCGGTGCA-3′; OC reverse primer 5′-CACTACCTCGCTGCCCTCC-3′; OC probe 5′-TGGACACAAAGGCTGCACCTTTGCT -3′. The probes were labeled at their 5'-ends with fluorescent dye FAM and at their 3'-end with the quencher dye TAMRA. The expression of housekeeping gene glyceraldehyde-3-phosphate-dehydrogenase (GAPDH) was quantified using »Human GAPD (GAPDH) TaqMan Universal Endogenous Control«, labeled with VIC/TAMRA (Applied Biosystems/Life Technologies). Gene expression levels were normalized according to the ΔΔCt method (Applied Biosystems/Life Technologies). Individual gene expression levels were first normalized to the expression level of GAPDH, and then to the expression levels of alveolar bone calibrator sample (AB1, explant culture), which was selected as a standard osteoblastic control to compare the –fold changes in expression levels between different patients.

### Osteogenic differentiation potential of alveolar bone cells

Alveolar bone cells of the early (second - third) and late (fifth - eight) passages were seeded into multiwell cell culture dishes at a density of 3.000 cells/cm^2^ and incubated for up to 3 weeks in the osteogenic medium (DMEM/F12, supplemented with 10% FBS, 50 μg/ml gentamicin and 0.08% Fungizone, 0.05 mM ascorbic acid 2-phosphate, 100 nM dexamethasone, 10 mM β-glycerophosphate) [34], with complete media changes on every 2–3 days. The control cultures were kept in parallel in the medium devoid of osteogenic supplements. The progression of osteogenesis was assessed after 1 week by histological staining for alkaline phosphatase activity, according to the manufacturer's instructions (Fast Blue RR Salt staining; Sigma-Aldrich). The mineralization potential of cultivated cells was assessed between days 10 and 21 (every 3–4 days) by von Kossa staining, which involves the substitution of silver for calcium in calcium salts. In brief, the cell cultures were fixed with 10% buffered formalin (pH neutral) for 30 min at 4°C, washed with deionized water and incubated in a 2% (w/v) silver nitrate solution for 15 min, in the dark. The cultures were then washed with deionized water, exposed to bright light for 30 min, and the presence of black mineral deposits was documented with light microscopy. The day when the cultures first stained positively was noted as a differentiation day (**Table 2**). Human bone marrow stromal cells (BMSCs, Lonza) were seeded and cultured in osteogenic and control media as differentiation controls, to compare the intensity of alkaline phosphatase activity and mineralization stainings.

**Table 2:** Mineralization potential of cultured alveolar bone cells.

	Positive staining of mineral at different passages and days of induction*			
Sample ID	Explant culture isolation		Collagenase isolation	
AB1	**P3**: day 13 +	/	**P3**: day 13 +	/
AB2	**P3**: day 14 +	/	**P2**: day 15 +	/
AB3	**P3**: day 16 +	/	**P3**: day 16 +	/
AB4	**P3**: day 13 +	/	**P3**: day 13 +	/
AB6	**P3**: day 18 +	/	**P3**: day 18 +	/
AB7	**P2**: day 18 +	**P6**: day 16 +	**P2**: day 18 +	**P6**: day 16 +
AB8	/	**P5**: day 16 +−	/	**P5**: day 16 +−
AB10	**P3**: day 20 +	**P7**: day 14 +	**P3**: day 20 +	**P7**: day 14 +
AB11	**P3**: day 19 +	**P7**: day 14 +	**P3**: day 20 +	**P7**: day 14 +
AB14	**P3**: day 13 +	**P8**: day 10 +	/	/
AB15	**P3**: day 12 +	**P7**: day 10 +	/	/
AB16	/	/	**P3**: day 14 +	/
AB17	**P4**: day 19 +−	/	/	/
AB18	/	/	**P2**: day 18 +−	/
AB19	/	**P6**: day 10 +	/	/

*Mineralization was evaluated semi-quantitatively in comparison to control cultures as shown in **Fig. 6**, and marked with symbols + (denoting mineralization), +− (denoting minimal mineralization) and – (denoting absence of mineralization). P stands for cell passage that was evaluated. D stands for day of induction that was evaluated. / Denotes that evaluation was not conducted.

### Statistical analyses

Statistically significant differences in cell yields were analyzed by Student's unpaired t-test and by linear regression analysis, using Microsoft Office Excel (Microsoft, Redmond, WA). Statistically significant differences in specific growth rates between cells isolated in explant cultures and those obtained after collagenase tissue digestion were analyzed by Student's paired t-test, using Microsoft Office Excel. Differences in gene expression among the cell culture passages were analyzed by Kruskal-Wallis test, followed by Dunn's post test for multiple comparisons, using the InStat statistical package (GraphPad Software, San Diego, CA). Differences in gene expression between cells isolated in explant cultures and those from collagenase tissue digestion were analyzed by Student's paired t-test, using Microsoft Office Excel. All differences with p<0.05 were considered statistically significant.

## Results

### Isolation and primary *in vitro* cultures of alveolar bone cells

In order to develop a reproducible cell harvesting procedure from alveolar bone samples, two tissue harvesting and two cell isolation procedures were tested. The use of bone forceps proved to be the more successful harvesting procedure for obtaining viable tissue as compared to bone drilling, resulting in growth of primary cells obtained from 28 out of the total of 30 samples (93%) from both mandibules and maxillae (**Table 1**). The use of bone drilling was less successful, as it resulted in growth of cells from only 2 out of 7 samples (29%), with both of them harvested from the maxillae (**Table 1**).

**Figure 1:**
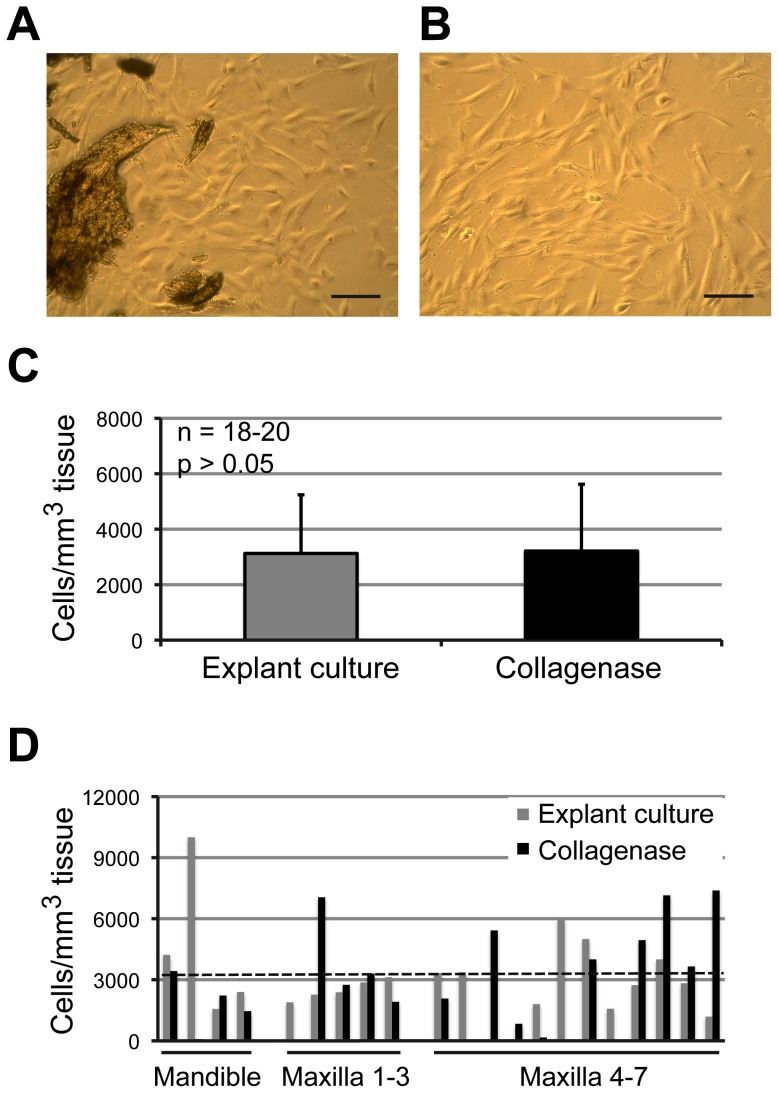
Isolation and yield of alveolar bone cells. Representative images of cells growing in explant cultures (n = 20) (A) and in collagenase digestion cultures (n = 18) (B) are shown. Scale bars: 100 μm. Average cell yields in confluent primary cultures were not significantly different between the two cell isolation techniques (p>0.05) (C). Cell yields varied greatly between the samples obtained from individual patients; no relationship between the cell yield and the site of tissue harvesting was observed (maxilla or mandible, tooth position numbers 1–3 or 4–7) (D). Dotted line indicates the average cell yield in all cultures (3.200 cells/mm^3^ of tissue).

**Figure 2:**
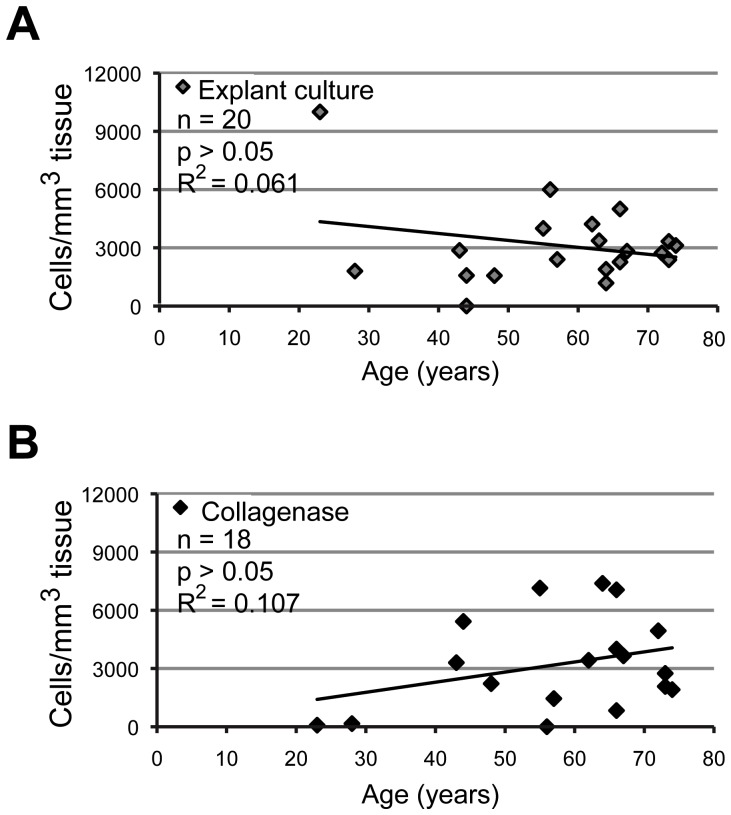
Cell yields in primary cultures as a function of patient age. Correlation coefficient (R^2^) values and significance levels (p) were calculated separately for explant cultures (n = 20) (A) and collagenase digestion cultures (n = 18) (B) by linear regression analysis. Cell yields did not correlate to the patient age.

For cell isolation and plating of the released cells, the bone explant culture preparation was compared to the stepwise collagenase digestion of bone tissue. Independent of the cell isolation procedure used, the cell growth was observed 1–3 days following the initiation of primary cultures. The cells were noted growing in small clusters after collagenase tissue digestion, or spreading from the bone explants on the surface of culture plates (**Figs. 1A, B**). Microscopic observations of the cultures showed the absence of residual bone marrow tissue after washing procedures. Primary cells proliferated on the plastic cell culture dish surface, reaching confluence in 9–22 days after initiation of the cultures. Average cell yields in confluent primary cultures were not significantly different between the explant (3.100 cells/mm^3^ tissue, n = 20) and collagenase digestion cultures (3.200 cells/mm^3^ tissue, n = 18, p>0.05) (**Fig. 1C**), and did not depend on either the site of tissue harvesting (**Fig. 1D**) or the age of the patients (p>0.05) (**Fig. 2**). As expected, the cell yields were variable among different patients (**Figs. 1** **,** **2**).

### Growth of alveolar bone cells in subcultures

**Figure 3:**
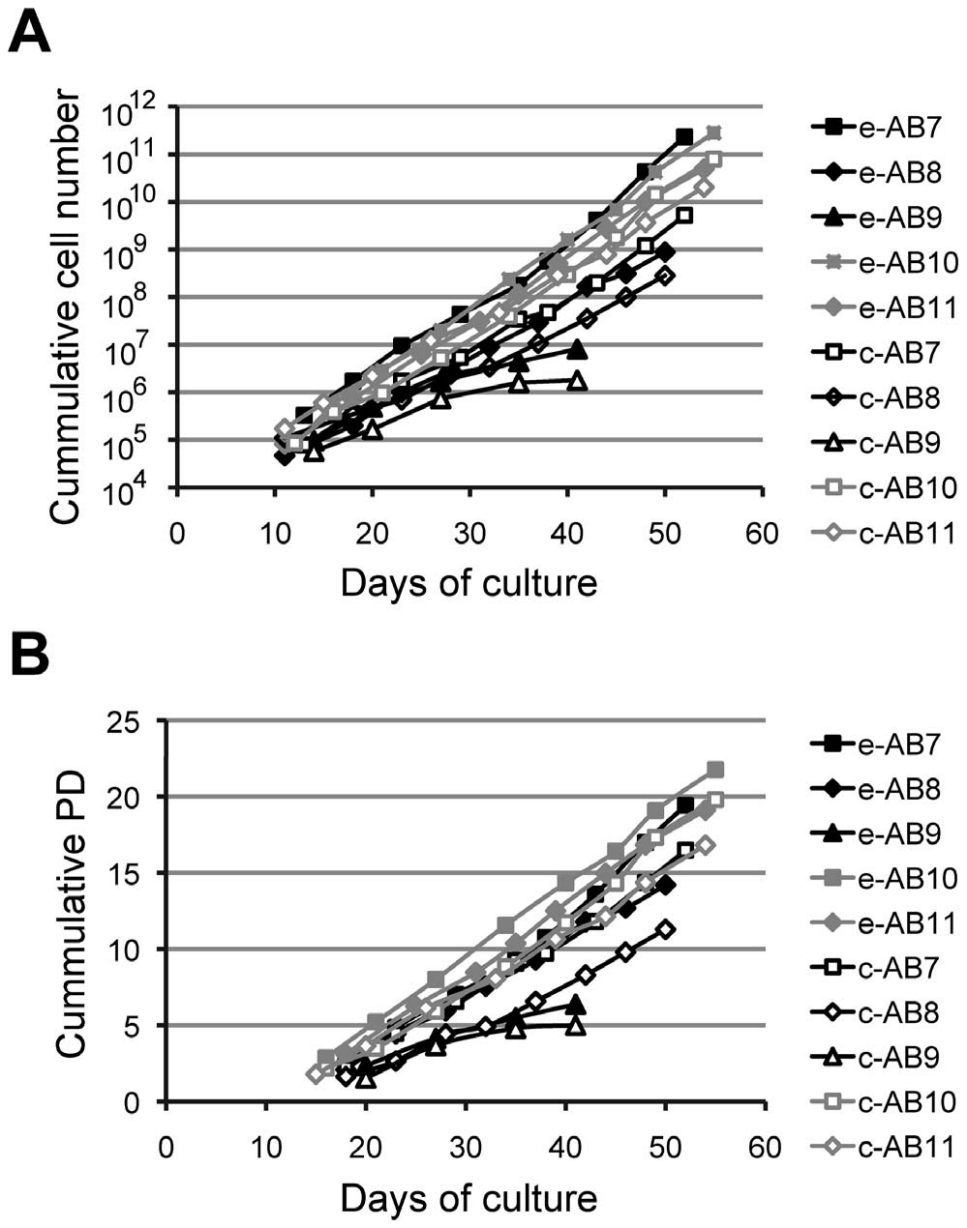
Growth of alveolar bone cells during the subculturing. Cells were isolated from 5 samples of alveolar bone in parallel by explant cultures (e-, full symbols) and by collagenase digestion (c-, open symbols) and were continuously subcultured for up to 8 subsequent passages. Increases in total numbers of cells (A) and in number of population doublings (PD) (B) are shown as a function of culture time, for each cell culture sample.

The potential of alveolar bone cells for longer *in vitro* proliferation was examined in 5 samples of alveolar bone, separately for the cells isolated by explant cultures and collagenase digestion. Alveolar bone cells from 4 samples (80%) exhibited sustained proliferation for eight subsequent passages (50–55 days of culture), with the total cell number increasing from approximately 10^6^ after 20 days of culture to over 10^10^ after 50 days of culture (**Fig. 3A**), reaching up to 22 additional population doublings after the primary culture (**Fig. 3B**). Proliferation rates were comparable for the cells isolated by the two techniques, as indicated by the slopes of the growth curves (**Fig. 3A**). Calculated average specific growth rate (μ) during passaging was 0.32/day for the collagenase digestion samples, and was slightly higher than the calculated average specific growth rate for the explant culture samples (0.27/day) (n = 4, p<0.05). The proliferating cells remained small and maintained fibroblastic morphology, exhibiting no morphological signs of senescence. In one of the tested samples (sample AB9, **Fig. 3**), the cell proliferation stopped at the fourth passage and the estimated maximum growth rate of cells decreased from 0.17/day at passage 1 to 0.02/day during passage 4.

### Gene expression of bone markers in cultured alveolar bone cells

**Figure 4:**
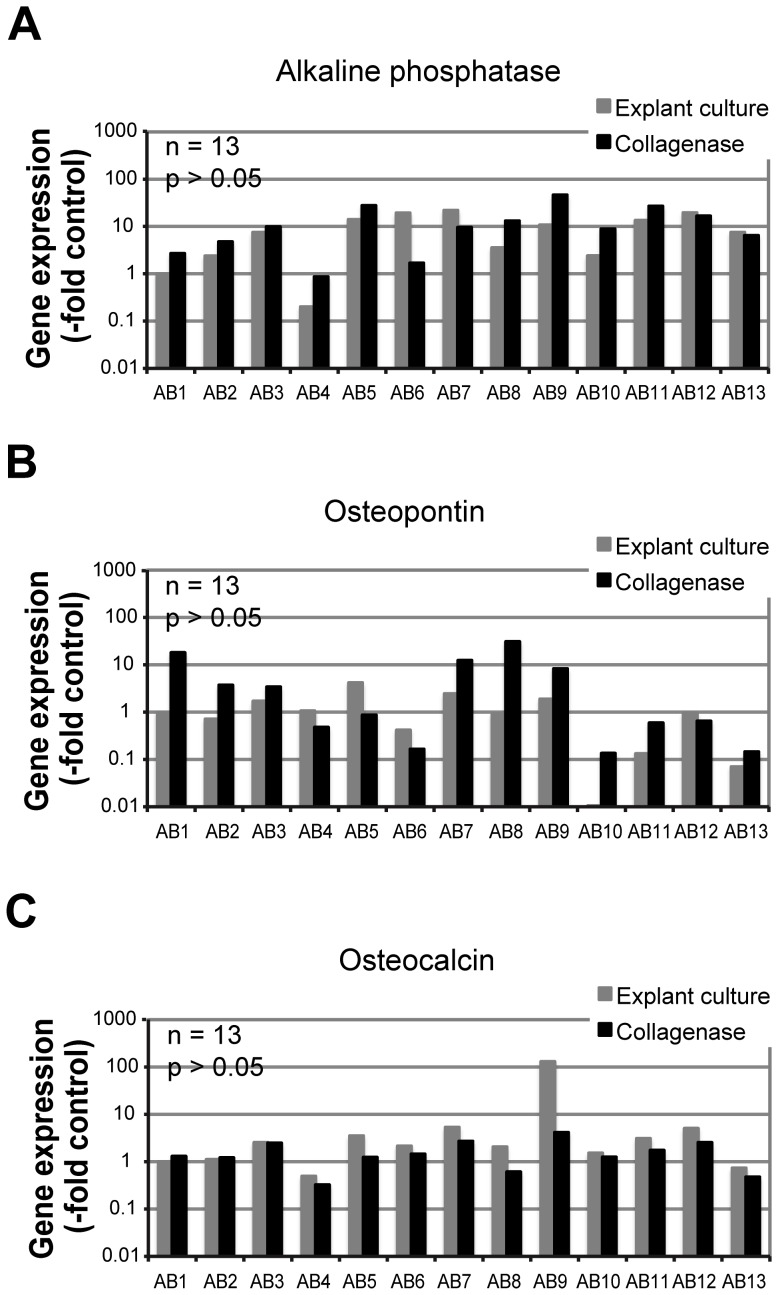
Relative gene expression levels of alkaline phosphatase (A), osteopontin (B) and osteocalcin (C). Alveolar bone cells isolated by explant cultures (grey bars) and by collagenase digestion (black bars) from 13 different patient samples were cultured separately and evaluated at the second passage. Data show expression levels of individual samples normalized to the osteoblastic control sample (-fold change). No significant differences in expression levels were found between cells from the two different isolation procedures (p>0.05).

In order to quantitatively evaluate the phenotype of alveolar bone cells during *in vitro* cultivation, mRNA expression of bone-specific markers alkaline phosphatase (AP), osteopontin (OP) and osteocalcin (OC) was analyzed. Consistent expression of all three bone markers was detected in all 13 samples of the second passage cells, isolated from alveolar bone by both explant culture and the collagenase digestion (**Fig. 4**). No significant differences could be observed in the mRNA expression profiles of cells obtained by the two different isolation procedures (n = 13, p>0.05). In some cases, the mRNA expression levels were higher in the explant culture-derived cells, whereas in others, higher mRNA expression was observed in cells derived from the collagenase digestion of bone tissue (**Fig. 4**). Relative levels of AP and OP mRNAs were more variable between the cells isolated from different patients (up to 100-times for AP, **Fig. 4A** and up to 400-times for OP, **Fig. 4B**, respectively) compared to mRNA expression levels of OC, which varied less than 10-times in all but one tested sample (**Fig. 4C**).

**Figure 5:**
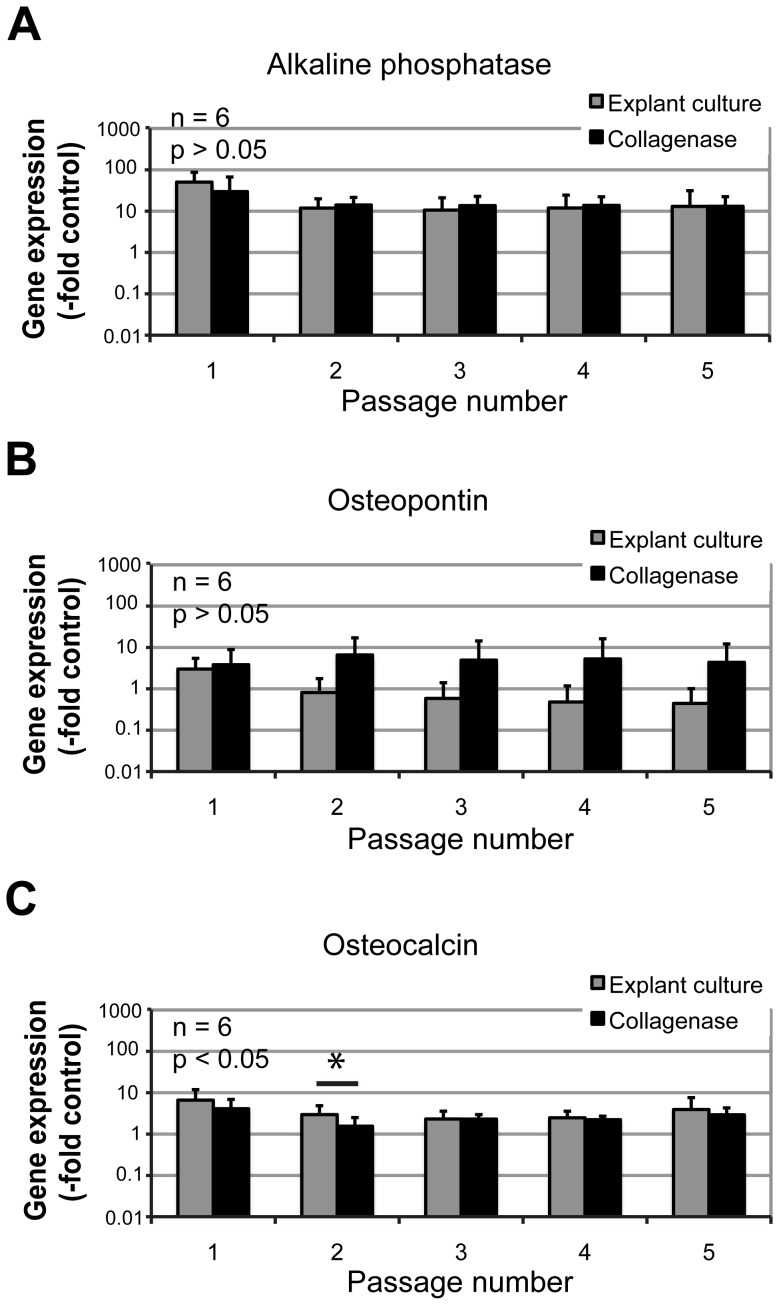
Relative osteogenic gene expression levels during *in vitro* culturing of alveolar bone cells. The cells were isolated by explant cultures (grey bars) and by collagenase digestion (black bars). Gene expression levels were normalized to the osteoblastic control sample and are shown as average –fold changes ± SD for 6 samples. *Denotes statistically significant difference between the explant cell culture and the collagenase digestion-derived cells (p<0.05). The differences between cell culture passages were not statistically significant (p>0.05).

Importantly, mRNA expression levels remained stable during five subsequent *in vitro* cell culture passages (n = 6) (**Fig. 5**). We noted slight decreases in bone markers expression after the first passage (most notably in the OP mRNA expression in collagenase-isolated cells), however these kinds of differences between passages were not statistically significant (p>0.05), and the expression levels remained stable afterwards. Similarly, bone marker mRNA expression levels were not significantly different between the cells isolated by explant cultures or collagenase digestion, with the only exception of small significant difference in OC mRNA expression detected at passage 2 (p<0.05).

### Osteogenic differentiation potential of cultured alveolar bone cells

**Figure 6:**
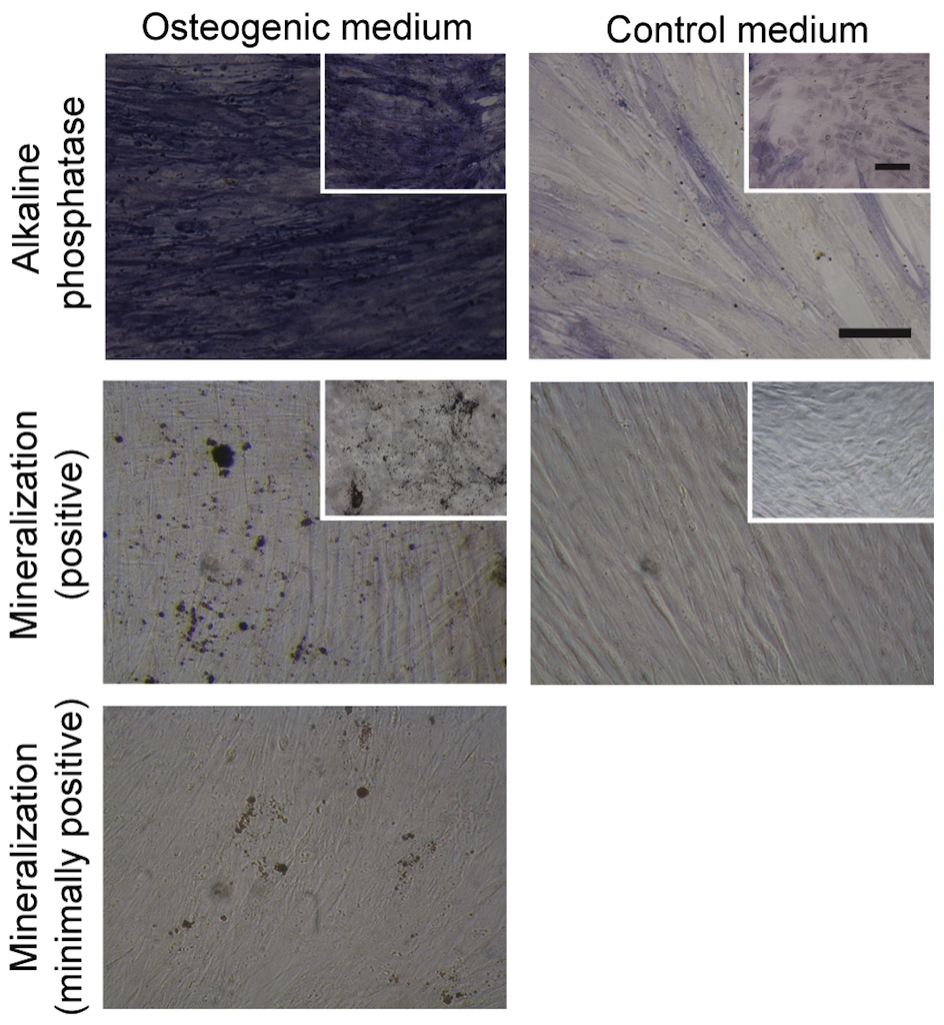
Differentiation and matrix mineralization of alveolar bone cells *in vitro*. Increased alkaline phosphatase (AP) activity (top left, purple stain) was noted following 1 week of cell culturing in osteogenic medium, as compared to a minimal AP activity detected in control medium (top right). During 2–3 weeks of differentiation in osteogenic medium, strong deposition (middle left, black deposits) or weaker deposition of mineralized matrix (bottom) was detected by von Kossa staining. No mineralized deposits were detected in control cultures (medium right). Images include representative samples. Insets show comparative BMSC cultures in osteogenic and control media. Scale bar (for all images): 50 μm.

Following quantitative gene expression analyses, the osteogenic potential of cultured alveolar bone cells was examined *in vitro* by assessing the activity of alkaline phosphatase and the extent of matrix mineralization. The induction of alveolar bone cells in monolayer cultures with osteogenic medium resulted in increased alkaline phosphatase activity after 1 week, as compared to the control cultures kept in a medium devoid of osteogenic factors (**Fig. 6**). During later phases of alveolar bone cells osteogenic differentiation (weeks 2–3) the accumulation of mineral deposits was noted (**Fig. 6**), with slight differences in the quantities of the mineralized matrix between the cells of different patients (n = 15) (**Table 2**). Comparable increases in alkaline phosphatase activity and matrix mineralization were noted in control BMSC cultures (**Fig. 6** **, Insets**). Importantly, the cells from early (second – fourth), as well as late cell culture passages (fifth–eighth), repeatedly exhibited positive staining of the mineralized matrix between second and third week of induction (**Table 2**). The mineralization potential was confirmed both for the cells grown in bone explant cultures, as well as for those isolated by collagenase digestion of bone tissue.

## Discussion

Our study was designed to assess human alveolar bone tissue as an autologous source of primary osteogenic cells for bone tissue engineering, as well as related drug and biomaterials testing applications. Based on prior work, our aim was to evaluate the parameters affecting routine isolation of human alveolar bone cells, and to evaluate the cell expansion potential and the maintenance of osteogenic phenotype during prolonged *in vitro* cultivation. Using a large number of primary alveolar bone samples (n = 37), we found that the use of bone forceps for tissue harvesting and the establishment of explant cultures resulted in reproducible growth of primary cells from tissue remnants obtained during periodontal surgical procedures. We found no effects of patient age, tissue harvesting site and cell isolation procedures on the cell yields and osteogenic marker expression. We were able to expand the cells to sufficiently large numbers (>10^10^ cells) for tissue engineering and related studies in up to eight consecutive cell culture passages. Importantly, we showed the maintenance of a stable osteogenic phenotype in expanded primary alveolar bone cells by using a combination of quantitative and qualitative assays of osteogenic markers, thereby excluding the possibility of cell dedifferentiation during culture. Phenotype maintenance in culture-expanded cells is critical for clinical application, and supports the use of alveolar bone cells as an *in vitro* model for drug testing or development of biomaterials.

The use of human tissue, which would normally be discarded during periodontal surgical procedures, presents a significant advantage for the preparation of TE-based bone products, as well as for obtaining primary cells for research studies. When preparing TE-products, such approach enables the surgeons to avoid inflicting additional injury to the patients and increasing the treatment costs due to additional surgical procedures needed for tissue harvesting. We used several washing steps before cell isolation, and added antimicrobials to washing solutions and culture media to prevent microbial contamination of the cultures. Despite using relatively small quantities of intra-operatively discarded bone tissues (**Table 1**), we were able to expand the cells to numbers that are sufficient for TE studies (**Fig. 3**) [5]. The protocols described here could easily be scaled-up in situations where larger quantities of bone tissue would be available for cell isolation.

We compared different procedures of tissue harvesting and cell isolation, as these were previously shown to influence the yield and phenotype of cultured osteogenic cells derived from various sources [18], [19], [20], [21]. In our experiments, bone drilling proved to be a less successful procedure of tissue harvesting (**Table 1**), possibly as a result of cell damage due to tissue overheating. It is known that elevated temperatures during surgical cutting lead to thermal necrosis and apoptosis of bone cells, as well as surrounding soft tissues. However, exposure of bone cells to temperatures lower than 47°C for short periods of time (< 1 min) was shown to induce expression of heat-shock proteins and increase the proliferation, differentiation and mineralization of osteoprogenitors [35], [36]. Therefore, adaptation of drilling regimes or using drills with cooling systems could be tested to optimize the tissue harvesting. Interestingly, the only 2 out of 7 samples obtained by drilling that exhibited cell growth were taken from maxillae, whereas the 5 remaining mandibular samples exhibited no cell growth. This difference was not observed when the bone tissue was harvested using bone forceps, where the majority of mandibular and maxillary samples resulted in a proper primary cell growth (**Table 1**).

No significant differences were observed in average cell yields between the two cell isolation procedures used. This is important, since bone tissue digestion with collagenase adds a significant cost to the cell preparation procedures for clinical and research studies. As expected, variability in numbers of isolated cells was noted among alveolar bone samples taken from different patients (**Fig. 1**). In this respect, our data are in agreement with previous studies where other osteogenic cell sources were evaluated and where interindividual variations in isolated cell numbers were also observed [37].

Fibroblastic morphology of cultured primary cells and the time required to start the growth and reach confluence were similar between the two cell isolation procedures used in our study, and were in agreement with previous reports [12], [24]. As in previous studies, we observed little or no cell growth around the cortical bone tissue, which was present in some samples and transferred to the culture dishes together with the isolated cells (data not shown) [24]. In our study, the site of tissue harvesting and the patient age were noted for each sample, but they could not be linked to significantly higher or lower cell yields in primary cultures, regardless of the cell isolation technique used. This is in contrast to some previous studies of mesenchymal stem/stromal cells isolated from the bone marrow, where a decline in cell numbers or proliferation potential was observed in older patients [38], [39]. To our knowledge, this is the first report evaluating the efficiency of different primary cell isolation procedures from a large number of human alveolar bone samples that have been harvested in different ways. Our data are in agreement with a previous study, where different culture media compositions were tested for the expansion of primary human alveolar bone osteoblasts up to 5 passages [27]. Importantly, we found that primary alveolar bone cells can successfully be isolated from tissue samples of elderly patients, which is again in agreement with previous reports [24], [27]. High proliferation potential of primary alveolar bone cells was expected, as the alveolar bone is constantly undergoing remodeling activity [40].

Previous studies also suggested a higher proliferation rate of early passage osteogenic cells isolated from the jaw bones in comparison to other, anatomically different bone tissue sources [10], [32]. In our study, most of the tested alveolar bone samples exhibited sustained *in vitro* proliferation during 8 passages, and reached >10^10^ cells, providing sufficient numbers for tissue engineering or *in vitro* testing studies (**Fig. 3**) [41]. We did not detect any morphological signs of senescence, and the cells maintained the characteristic fibroblastic morphology and high growth rates during passaging (**Figs. 1** **,** **3**) [42]. In comparison, primary bone marrow stromal/stem cell populations derived from the iliac bone required growth factor supplementation (for example the basic fibroblast growth factor) or the use of selected fetal bovine serum lots to sustain their *in vitro* growth at comparable rates to those exhibited by alveolar bone cells in our study [10], [22]. Therefore, media supplemented with appropriate growth factors could be tested to enhance the growth of alveolar bone cells in rare cases where they exhibit low proliferation rates (**Fig. 3**).

Since primary cells are prone to dedifferentiation during their *in vitro* expansion [22], [43], we continuously monitored the cell phenotype and differentiation potential during 8 consecutive culture passages. In prior studies of osteogenic cell sources, expression of mesenchymal surface antigens was commonly evaluated by flow cytometry [24], [41]. However, the high expression of mesenchymal markers did not necessarily correlate with differences in the cell differentiation potentials. In the current study, we chose to evaluate the cells using a combination of molecular assays of osteogenic markers and *in vitro* differentiation assays. We first quantitatively evaluated the mRNA levels of three commonly studied bone markers, AP, OP and OC, that are expressed during osteogenic differentiation and maturation of osteoblasts [42]. We separately evaluated second passage cells that were isolated by explant cultures and collagenase digestion from 13 samples, and found consistent expression of osteogenic markers in all samples, characterized by relatively high interindividual variations (**Fig. 4**). Interestingly, gene expression levels of OC were the most conserved between the samples, whereas the variations of AP and OP gene expression levels were larger. The mRNA expression levels of AP and OP are known to change dynamically during osteogenic differentiation [44], therefore the differences observed among individual samples could potentially reflect such changes within the isolated cell populations. Importantly, the osteogenic gene expression levels were sustained during five cell culture passages (**Fig. 5**), regardless of the cell isolation procedure used, suggesting that the cultured cells retain their characteristic phenotype. It would be interesting to extend these studies to a larger number of genes related to bone development, and to proteomic analyses, to potentially identify an expression signature characteristic for alveolar bone cells [45], [46].

Finally, we tested the potential of expanded alveolar bone cells to form mineralized bone matrix, using a standard osteogenic differentiation assay in monolayer cultures and BMSCs as differentiation controls. We observed an increase in AP activity after 1 week of osteogenic induction (**Fig. 6**), in agreement with previous studies of primary human bone cells [24], [25], [42]. The increased AP activity was followed by matrix mineralization, as shown by the positive von Kossa staining of the cultures after 2–3 weeks (**Fig. 6**, **Table 2**), again in agreement with the reported dynamics of osteogenic differentiation [24], [25], [42]. We used a semi-quantitative assay to assess matrix mineralization in order to evaluate a large number of cell samples from different cell isolation procedures, culture passages, and duration of osteogenic induction. In this way, the persistence of functional mineralization potential in cells of late (fifth–eight) passages from several patient' samples was confirmed. In addition, a similar level of mineralization was observed in samples that were tested repeatedly at early and late cell culture passages, further confirming the maintenance of functional potential (**Table 2**). These data are in agreement with previous studies, which demonstrated osteogenic potential of alveolar bone cells in monolayer and three-dimensional cultures by increased AP activity and increased gene and protein expression of several osteogenic markers [10], [24], [25], [26], [28], [32]. For clinical translation, we previously showed that dynamic culture of TE-constructs, prepared by seeding alveolar bone cells on hydroxyapatite granules in fibrin glue enhanced the expression of osteogenic phenotype compared to monolayer cultures [25]. In addition, preliminary results from our pilot clinical trial suggested accelerated regeneration of periodontal bone defects after the transplantation of autologous alveolar bone cells on hydroxyapatite granules [6].

The current study was performed as the basis for large-scale preparation of TE-bone substitutes and suggests that primary alveolar bone represents a potential cell source that could enhance the regeneration of periodontal bone defects. With clinical application in mind, we limited the cell characterization studies to <8 culture passages, when the cultures reached sufficient cell numbers for the preparation of TE-bone substitutes [41]. Currently, most clinical study protocols involve the use of early (<5) passage cells, to minimize the changes associated with *in vitro* cultivation [29], [30], [31], [47], [48], [49]. However, to fully utilize the primary alveolar bone cells as an *in vitro* model, the phenotype and proliferation of cells in later passages should be evaluated in future studies.

In contrast to several clinical reports where periosteal cells were harvested specifically for the purpose of TE-bone substitutes preparation [31], [47], [48], [49], our approach involves the use of bone tissue remnants, obtained during routine periodontal surgical procedures, as a source of osteogenic cells, thus avoiding additional injury to patient due to tissue harvesting. A few prior studies reported clinical application of TE-bone substitutes derived from alveolar bone cells [29], [30], [31], [50]. Pradel and colleagues used second passage mandibular and maxillary osteoblasts, isolated by establishing explant cultures and cultured on collagen scaffolds 3–4 days, to regenerate bone after mandibular cyst enucleation and for osteoplasty in patients with cleft alveolus [29], [30]. After 6 months, bone regeneration was comparable between the tissue engineering group and the control autologous iliac bone transplant group. Springer and colleagues used first passage maxillary osteoblasts, isolated by establishing explant cultures and cultured on deproteinized bovine bone scaffolds *in vitro* for 1.5 months, as one of the groups in sinus augmentation procedures [31]. New vital bone formation with sufficient stability for implant placement was found with osteoblast-derived TE-substitutes, similarly to periosteal cells-derived TE-substitutes. Mangano and colleagues used third/fourth passage mandibular bone marrow osteoblasts, isolated from bone cores by collagenase digestion and cultured on polymer scaffolds for 1 week, for sinus augmentation procedures [50]. After 6 months, clinical, histological and computed tomography evaluations revealed a significant average vertical bone gain. However, mineralized tissue density was considerably higher in the hydroxyapatite scaffold-only control group. Together with our work, these studies suggest the clinical potential of alveolar osteoblast-derived TE-bone substitutes. However, large differences in cell culture and tissue engineering protocols, including cell isolation and cultivation procedures, cell passage numbers and biomaterials used for tissue engineering, prevent general conclusions. As suggested by the interindividual variability of our results, any future clinical studies would need to involve parallel characterization of the specific cell preparations used in the studies, in order to better understand the treatment outcomes. In addition, future studies are needed to compare the efficacy of different cell types for periodontal bone regeneration, in relation to their tissue origin and procedures used for the TE-bone substitute preparation.

## Conclusions

In conclusion, our study demonstrates the possibility to reproducibly isolate and expand primary human alveolar bone cells from bone tissue samples remaining at periodontal surgical procedures, using a combination of bone forceps for tissue harvesting and explant culture initiation for primary cell isolation. We found that primary human alveolar bone cells maintained constant growth potential *in vitro* over 8 cell culture passages, thereby allowing their expansion to numbers that are sufficient for tissue engineering and *in vitro* testing studies. Importantly, our data also showed that the cells maintained the osteogenic phenotype during *in vitro* cultivation for over 5 cell culture passages. Based on our results, we suggest that the primary human alveolar bone cells represent a suitable cell source for bone tissue engineering, as well as a qualified experimental model for *in vitro* studies related to periodontal regeneration and testing of various drugs and biomaterials.
